# Monitoring and assessing the quality of care for youth: developing an audit tool using an expert consensus approach

**DOI:** 10.1186/s13033-015-0019-5

**Published:** 2015-07-13

**Authors:** Stefanie Puszka, Tricia Nagel, Veronica Matthews, Diana Mosca, Rebecca Piovesan, Annapurna Nori, Ross Bailie

**Affiliations:** Menzies School of Health Research, PO Box 41096, Casuarina, NT 0810 Australia; One21seventy, The National Centre for Quality Improvement in Indigenous Primary Health Care, PO Box 10639, Adelaide Street, Brisbane, QLD 4000 Australia; Watto Purrunna Aboriginal Health Service, Northern Adelaide Local Health Network, SA Health, 1 Gilles Crescent, Hillcrest, SA 5086 Australia

**Keywords:** Clinical audit, Quality improvement, Quality indicators, Standard of care, Adolescent health services, Primary healthcare, Indigenous health

## Abstract

**Background:**

The mental health needs of young people are often inadequately met by health services. Quality improvement approaches provide a framework for measuring, assessing and improving the quality of healthcare. However, a lack of performance standards and measurement tools are an impediment to their implementation. This paper reports on the initial stages of development of a clinical audit tool for assessing the quality of primary healthcare for Australian Indigenous youth aged 12–24 including mental health services provided within primary care.

**Methods:**

Audit items were determined through review of relevant guidelines, expert reference group consensus opinion and specific inclusion criteria. Pilot testing was undertaken at four Indigenous primary healthcare services. A focus group discussion involving five staff from a health service participating in pilot testing explored user experiences of the tool.

**Results:**

Audit items comprise key measures of processes and outcomes of care for Indigenous youth, as determined by the expert reference group. Gaps and conflicts in relevant guidelines and a lack of agreed performance indicators necessitated a tool development process that relied heavily on expert reference group advice and audit item inclusion criteria. Pilot testing and user feedback highlighted the importance of feasibility and context-specific considerations in tool development and design.

**Conclusions:**

The youth health audit tool provides a first step in monitoring, assessing and improving the way Indigenous primary healthcare services engage with and respond to the needs of youth. Our approach offers a way forward for further development of quality measures in the absence of clearly articulated standards of care.

## Background

### Youth health services

Youth is a critical period of development and transition in which behaviours and habits can be established, experimentation and risk taking may take place and mental health problems can emerge. From the perspective of health services, this important life phase presents an opportunity for facilitating healthy lifestyles and identifying and mitigating risk factors for future ill-health [1]. However, there are many barriers preventing young people from accessing health services, such as healthcare availability, accessibility, acceptability and equity [2].

### The role of primary healthcare

Primary healthcare (PHC) provides the foundation of healthcare systems internationally through key roles in prevention and early intervention [3, 4]. In some remote or developing contexts, PHC services may be the main or only healthcare providers. The frequent comorbidity of depression with substance misuse [5] and chronic disease [6] makes PHC an ideal avenue for prevention and management of these conditions. Amongst PHC services internationally, there is a need to establish and monitor standards of care for youth [2].

### Australian Indigenous youth health

Young Indigenous Australians have a higher burden of disease than other Australian youth, largely attributable to high rates of mental illness such as anxiety and depression, substance use and injuries [7]. In comparison to their non-Indigenous counterparts, Indigenous young people also suffer from substantially higher rates of chronic conditions, including diabetes, hearing loss, skin diseases, rheumatic heart disease [8], poor sexual health [7] and experience considerable health challenges across a range of other indicators [7].

### Healthcare standards for Australian Indigenous youth

Indigenous youth under-utilise health services and engage with healthcare at more advanced stages of illness and for shorter periods in comparison to non-Indigenous youth [9, 10]. The quality, capacity and cultural appropriateness of health services have been recognised as key factors in engagement with services and in health outcomes for Indigenous youth [7]. However, despite the different health profile of Indigenous youth and unlike some other areas of Indigenous health, systems for monitoring and improving the performance of Indigenous youth health services are limited. There is no framework specific to Indigenous youth and the indicator framework for Australian youth health does not provide data in a format that readily facilitates health system improvements, combining primary healthcare and hospital data. Minimal data are available to monitor standards at jurisdictional, regional or healthcare centre levels [11].

A need for data to help facilitate health service system improvements which are locally-relevant, culturally appropriate and responsive to the needs of Indigenous youth has been identified from within the Indigenous primary healthcare sector [12]. We were not able to identify any tools for monitoring the performance of primary healthcare services in Indigenous youth health in a systematic search. Our aims were therefore to develop an audit tool for monitoring and assessing the delivery of health and wellbeing services for Indigenous youth for use within a national quality improvement program.

### Quality improvement as an approach to improving service delivery

**Figure 1:**
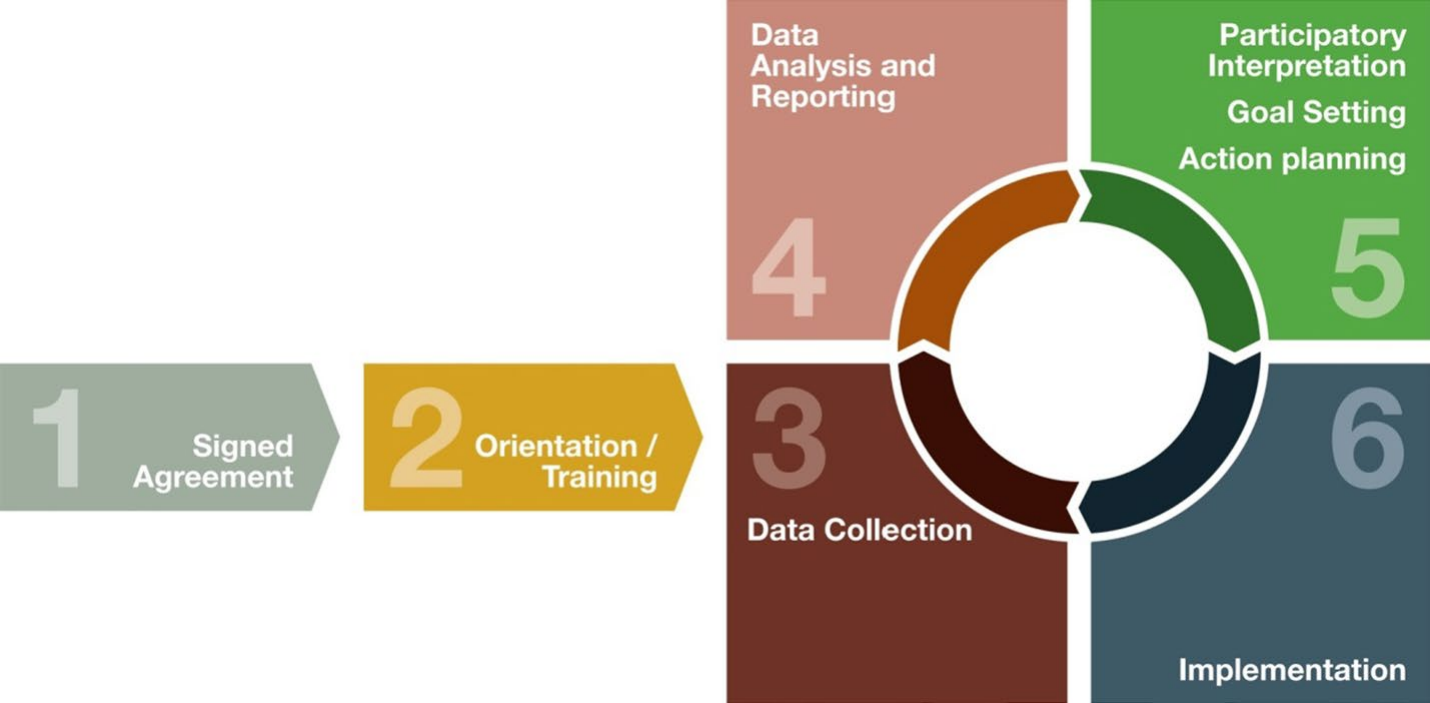
One21seventy quality improvement cycle.

Modern continuous quality improvement uses objective information to analyse and improve health systems, processes and outcomes [13, 14]. Through our Audit and Best Practice for Chronic Disease projects, we have used a quality improvement approach to develop tools and processes to facilitate cycles of feedback reports and workshops, goal setting, action planning and implementation of systems changes within Indigenous primary healthcare services (Figure 1) [15]. Over 230 Indigenous health centres across Australia have participated in evidence-based quality improvement processes under the auspices of One21seventy—the National Centre for Quality Improvement in Indigenous Primary Health Care. The One21seventy approach to quality improvement has been reported elsewhere [16–18].

### Quality improvement indicator development

Donabedian [19] provides a theoretical approach to measuring quality of healthcare based on structures, processes and outcomes of care, which has been generally accepted by quality improvement practitioners. Traditionally, the development of quality indicators has involved a combination of evidence-based guidelines and expert consensus. However, few published pilot and validation studies of audit tools contain explicit information about the methodology used to develop indicators or the assessment criteria used to evaluate them [20].

In a systematic review of guideline-based quality indicator development methods, Kötter, Blozik and Scherer [21] found that no gold standard for developing quality indicators exists. Two broad approaches to identifying indicators from guidelines were identified: the extraction of all guideline recommendations and the selection of indicators using a systematic expert consensus approach; and the selection of a limited number of guideline recommendations as the basis for indicators. In studies where a limited selection of guideline recommendations were extracted, criteria for preselection included the size of the impact on patient health; the relevance to obtaining value for money; the importance to quality of healthcare provided; the feasibility of monitoring; the level of evidence; the grade of the recommendation; and measurability.

According to Baker and Fraser [22], quality indicators should be determined according to the strength of the evidence behind best practice guidelines and their potential influence on outcomes of care. They should include diagnostic and management indicators and establish realistic standards of care; however this approach does not take into account the burden of disease or stakeholder engagement. In contrast, Bickman [23] has advocated an approach based on program logic, involving the mapping of all stages of health service delivery processes to develop process-based quality indicators. The interaction of this method with evidence-based practice is unclear.

## Aims

This paper describes the initial stages of development of a tool for auditing primary healthcare services for Indigenous youth aged 12–24. The audit tool is designed for use by clinical and non-clinical primary healthcare service staff to audit client records and includes scheduled mental health services provided within primary care. Specific objectives are to describe:

- The development of audit items using a systematic methodology of identification of relevant items from guideline review and expert consultation; and
- How results from pilot testing and a user feedback process informed the further development of the audit tool.

## Methods

**Figure 2:**
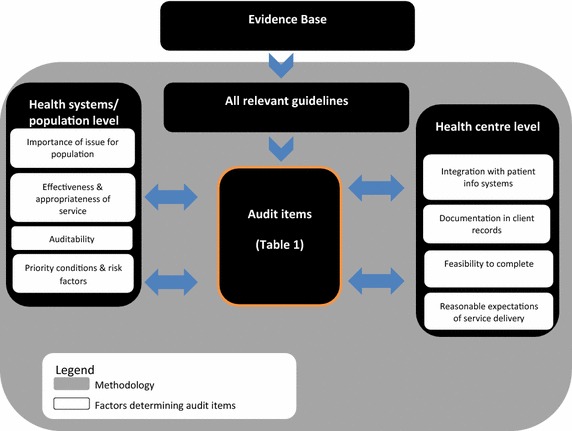
Audit tool development methodology.

Our methodology is consistent with an approach described by Kötter et al. [21] where standards of care have not been pre-defined. In this method, evidence-based guideline recommendations are identified and audit items developed using a systematic expert consensus approach and explicit selection criteria. The youth health audit tool was developed through a process that considered factors relevant to the health system or population and health centre levels (Figure 2). An Expert Reference Group (ERG) was formed to advise on the development of a draft document. Findings from pilot testing and a user feedback process informed further refinement in addition to consultation with the ERG.

### Establishment of expert reference group

**Figure 3:**
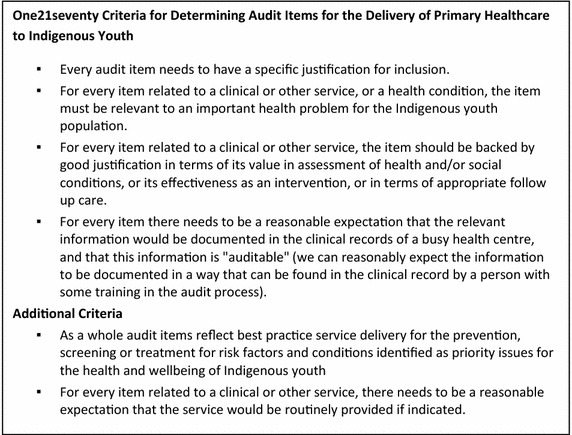
Criteria for determining audit items for the delivery of primary healthcare to Indigenous youth.

The ERG determined content validity of the tool [24] based on expert consensus opinion. The ERG comprised expertise in Indigenous youth health from primary healthcare services and research organisations with representatives from four states and territories. The group met on a monthly basis over an 18 month period via teleconference and was facilitated by a member of the research team. Terms of reference for the group were developed and agreed, including standard One21seventy criteria for determining audit items (with minor changes for the Indigenous youth population). Initial inclusion criteria were based on the importance of the health issue for the Indigenous youth population; effectiveness and appropriateness of the assessment, intervention or service; and ‘auditability’ or expectation of finding relevant information in client records and reporting on the item (Figures 2, 3).

Group facilitation techniques were used to achieve a consensus amongst ERG members. In instances where members disagreed on the inclusion of a specific item in the audit, all members were invited to outline and justify their views. Generally, disagreements were resolved with reference to the inclusion criteria, and particularly the need to satisfy the ‘auditability’ clause.

### Development and refinement

Relevant guidelines for Indigenous youth health were identified (Table 1) and used as a basis for development of initial audit items. For example, a national Indigenous preventive health guidelines recommendation to assess smoking status as part of a periodic health check provided the basis for an audit item to measure the proportion of clients with assessment of smoking status over the previous 24 months [25].

**Table 1:** Guidelines for youth health in Indigenous primary healthcare

Topic	Guidelines
General	National Guide to a Preventive Health Assessment in Aboriginal and Torres Strait Islander Peoples [25]
	MBS Health assessment for Aboriginal and Torres Strait Islander people (MBS Item 715) [26]
	Central Australian Rural Practitioners Association Standard Treatment Manual [27]
	Minymaku Kutju Tjukurpa—Women’s Business Manual [28]
	Queensland Health and the Royal Flying Doctor Service Queensland Section Primary Clinical Care Manual [29]
Sexual health	Minymaku Kutju Tjukurpa—Women’s Business Manual [28]
	Queensland Sexual Health Clinical Management Guidelines [30]
	National Management Guidelines for Sexually Transmissible Infections [31]
	WA Department of Health Guidelines for Managing Sexually Transmitted Infections [32]
Alcohol and other drugs	Alcohol Treatment Guidelines for Indigenous Australians [33]
	Clinical Guidelines and Procedures for the Use of Methadone in the Maintenance Treatment of Opioid Dependence [34]
	National Clinical Guidelines for the Use of Buprenorphine in the Treatment of Opioid Dependence [35]
Processes of care	Interpretive Guide of the RACGP Standards for General Practice for Aboriginal and Torres Strait Islander Health Services [36]
Others	Recommendations for Clinical Care Guidelines on the Management of Otitis Media in Aboriginal and Torres Strait Islander Populations [37]
	Diagnosis and management of acute rheumatic fever and rheumatic heart disease in Australia—an evidence-based review [38]
	National Physical Activity Guidelines [39]
	Queensland Chronic Disease Guidelines [40]

### Pilot testing

The performance of the tool was pilot tested at four health centres in urban, regional and remote communities in the Northern Territory, South Australia, Western Australia and Queensland. The health centres included a range of organisation sizes and governance arrangements. At each pilot site, 30 client records were audited by trained auditors and health service staff. Pilot testing aimed to assess the recording in client records of specific items of care and the ease of use of the audit tool within different paper-based and electronic patient information systems. Notes and feedback from auditors were used to assess performance.

### User feedback

Staff taking part in the pilot at one site participated in a semi-structured focus group discussion to explore user experiences of the tool. Staff at this health service were undertaking a number of complementary projects relating to Indigenous youth health and two were represented on the ERG. Focus group participants were required to have completed at least one youth health audit using the audit tool. Purposive and maximum variance sampling [41] were employed to provide representation of a range of roles and organisational levels. Five staff including Indigenous and non-Indigenous participants (a manager, two project officers and two quality improvement coordinators) took part in a discussion of approximately 1 h duration at the health service. The focus group was facilitated by the first author using an interview guide. Consent was obtained from all participants through a written agreement. The focus group discussion was audio recorded, transcribed and distributed to participants for checking and confirmation. Results were reported to the ERG and contributed to further refinement of the audit tool. Ethics approval was provided by ethics committees in each jurisdiction where the tool was piloted and qualitative data was gathered.

### Qualitative analysis

Following a period of reflection, data were broken into units of meaning and initial themes were identified and discussed by two members of the research team. Initial manual data coding was conducted and the frequency of each theme’s appearance and its distribution across the range of participants determined to identify major and minor themes. Initial themes were then re-assessed and final themes were agreed by the research team and applied to the data through coding. A process of thematic analysis was undertaken to consider and interpret findings. Participants were consulted about initial findings and given the opportunity to contribute to further analysis through circulation of a draft manuscript.

## Results

### Audit tool content

The audit tool comprises items under the headings: general information; attendance at health centre; recording of key health information; scheduled immunisations; protective factors, risk factors, brief interventions and referral; scheduled services and follow up. A key component is a section on psychosocial concerns based on the HEEADSS (Home environment, Education and employment, Eating and diet, Activities and peer relationships, Drugs and alcohol, Sexual behaviour risks, Suicide and emotional wellbeing) assessment, an internationally accepted youth psychosocial assessment tool recommended by national guidelines for Indigenous youth [25, 41]. Cultural engagement and gambling were added to the HEEADSS items following recommendation from the ERG. Other items added to the audit tool following ERG advice were assessments of mature minor status and client consent. Audit items assess processes of care (including documentation of key client information), or a combination of processes and outcomes (for example, combining assessment of client risk status and the result of the risk assessment) (Table 2); assessment of the structure of care is dealt with separately in the One21seventy process through systems assessment (Figure 1).

**Table 2:** Final set of audit items in the youth health audit tool

Audit Item	Measure type	Likely documentation in client record	Frequency of reporting
General Information			
Current Medicare number recorded	Process (documentation)	Client summary	On first presentation
Date of birth	Process (documentation)	Client summary	On first presentation
Sex	Process (documentation)	Client summary	On first presentation
Indigenous status	Process (documentation)	Client summary	On first presentation
Mature minor assessment	Process (client centred)	Service items	First time client presents without parent/guardian
Attendance			
Date last attended	Process (service delivery)	Client summary	Routinely
Follow-up if no attendance within 24 month	Process (service delivery)	Client summary	Every 24 months
Reason for last attendance	Outcome	Client summary	Routinely
Seen by Aboriginal Health Practitioner	Process (client centred)	Events	Routinely
Documentation of other regular primary healthcare services attended	Process (documentation)	Service items or progress notes	As required
Key health information			
Documented long term health conditions	Process (documentation)	Client summary	On diagnosis
If yes, documentation of a management plan	Process (service delivery)	Progress notes	Every 24 months
Evidence of child/adult health check (MBS 715)	Process (service delivery)	Medicare billing	Annually
Evidence of alternative child/adult health check	Process (service delivery)	Client summary	Annually
Evidence of youth health check	Process (service delivery)	Client summary	Annually
Consent for health check	Process (client centred)	Service item	As part of annual health check
Immunisations			
Completed immunisation chart/record	Process (service delivery)	Immunisations	As indicated
Hep B	Process (service delivery)	Immunisations	As indicated
HPV	Process (service delivery)	Immunisations	As indicated
VZV	Process (service delivery)	Immunisations	As indicated
DTPa	Process (service delivery)	Immunisations	As indicated
Influenza	Process (service delivery)	Immunisations	As indicated
Pneumococcal	Process (service delivery)	Immunisations	As indicated
Protective factors, risk factors, brief interventions and referral			
Tobacco use	Process/outcome	Risks or service items	As part of annual health check or opportunistically
Tobacco use actions taken	Process (service delivery)	Risks or service items or recalls	As indicated
Tobacco use actions reviewed (within 3 months)	Process (service delivery)	Risks or service items or recalls	As indicated
Tobacco use report received from referral agency (within 6 months)	Process (service delivery)	Risks or service items or recalls	As indicated
Alcohol use	Process/outcome	Risks or service items	As part of annual health check or opportunistically
Alcohol use screening tools	Process (documentation)	Risks or service items	As part of annual health check or opportunistically
Alcohol use actions taken	Process (service delivery)	Risks or service items or recalls	As indicated
Alcohol use actions reviewed (within 3 months)	Process (service delivery)	Risks or service items or recalls	As indicated
Alcohol use report received from referral agency (within 6 months)	Process (service delivery)	Risks or service items or recalls	As indicated
Other substance use	Process/outcome	Risks or service items	As part of annual health check or opportunistically
Other substance use screening tools	Process (documentation)	Risks or service items	As part of annual health check or opportunistically
Other substance use actions taken	Process (service delivery)	Risks or service items or recalls	As indicated
Other substance use actions reviewed (within 3 months)	Process (service delivery)	Risks or service items or recalls	As indicated
Other substance use report received from referral agency (within 6 months)	Process (service delivery)	Risks or service items or recalls	As indicated
Sexual activity and risk discussion	Process/outcome	Service item or progress notes	As part of annual health check or opportunistically
Sexual risk behaviour actions taken	Process (service delivery)	Service items or recalls	As indicated
Sexual risk behaviour actions reviewed (within 3 months)	Process (service delivery)	Service items or recalls	As indicated
Sexual risk behaviour report received from referral agency (within 6 months)	Process (service delivery)	Service items or recalls	As indicated
Mental health assessment	Process/outcome	Service items	As part of annual health check or opportunistically
Mental health assessment screening tools	Process (documentation)	Service items	As part of annual health check or opportunistically
Self harm and suicide discussion if risk factors present	Process (service delivery)	Service items	As indicated
Mental health, self harm or suicide risk actions taken	Process (service delivery)	Service items or recalls	As indicated
Mental health, self harm or suicide risk actions reviewed (within 3 months)	Process (service delivery)	Service items or recalls	As indicated
Mental health, self harm or suicide risk report received from referral agency (within 6 months)	Process (service delivery)	Service items or recalls	As indicated
Lifestyle discussion	Process (service delivery)	Risks or service items	As part of annual health check or opportunistically
Scheduled services			
BMI for age and gender	Process/outcome	Service items	As part of annual health check
Waist circumference	Process/outcome	Service items	As part of annual health check
Weight and waist actions taken	Process (service delivery)	Service items or recalls	As indicated
Oral health check	Process/outcome	Service items	As part of annual health check or symptomatically
Oral health action taken	Process (service delivery)	Service items or recalls	As indicated
Ear/hearing check	Process/outcome	Service items	As part of annual health check or symptomatically
Ear health/hearing actions taken	Process (service delivery)	Service items or recalls	As indicated
Cardiac auscultation (heart check)	Process/outcome	Service items	As part of annual health check or symptomatically
Heart health actions taken	Process (service delivery)	Service items or recalls	As indicated
Skin exam	Process/outcome	Service items	As part of annual health check or symptomatically
Skin health actions taken	Process (service delivery)	Service items or recalls	As indicated
Blood pressure	Process/outcome	Service items	As part of annual health check
Blood glucose test	Process/outcome	Service items	As part of annual health check
Blood glucose action taken	Process (service delivery)	Service items or recalls	As indicated
Pap smear	Process/outcome	Service items	As part of annual health check
Pap smear action taken	Process (service delivery)	Service items or recalls	As indicated
Sexual health check	Process/outcome	Service items	As part of annual health check or symptomatically
Sexual health actions taken	Process (service delivery)	Service items or recalls	As indicated

**Figure 4:**
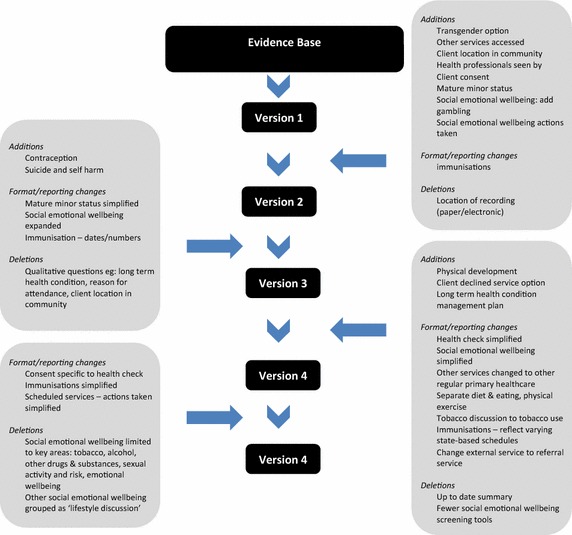
Audit tool development process.

The audit tool underwent four major phases of development as the ERG considered an initial draft and results from pilot testing and user feedback processes (Figure 4). Time taken to complete the audit and user feedback on the length of the tool provided impetus to further prioritise audit items. The original One21seventy criteria for determining the inclusion of audit items (Figure 3) required refinement for the purpose of the youth health audit tool. Further prioritisation of audit items was determined by the ERG according to best practice service delivery for the prevention, screening or treatment for risk factors and conditions identified as priority issues for the health and wellbeing of Indigenous

youth and considerations of what could be reasonably expected of health service practice and documentation, in addition to the original criteria (Figure 2). As a result, some psychosocial items were included in a briefer format and grouped as a ‘lifestyle discussion’, which included home environment, education, employment, activities and peer relationships, diet and eating, gambling and cultural engagement; and in the scheduled services section, items relating to follow up of abnormal findings were grouped as ‘actions taken’. Some other items that were not well documented in client records and which the ERG advised were unlikely to be specifically documented were removed, including discussion of contraception and physical development.

Gaps and conflicts in relevant guidelines for Indigenous youth health and a lack of agreed standards of care necessitated revision. In the final version of the tool, the ERG agreed to follow national Indigenous preventive health guidelines [25]. A lack of standard terminology of components of care delivery, particularly relating to psychosocial issues such as brief interventions and social or cultural treatments required further consideration, discussion and advice from the ERG and specification in the protocol.

### Pilot testing

The audit tool was generally found to be straightforward to use within different paper-based and electronic patient information systems. Ease of use was facilitated where health services had adopted standard ways and locations for recording information in client files across practitioners. It was less time consuming to complete in health services with standard recording protocols, in services which stored client information only in electronic systems (without supplementing with paper-based records) and where electronic systems included a specific tab for recording psychosocial information.

### User feedback

#### Scope and content

A major theme of the focus group discussion was the scope and content of the audit tool. While there was a consensus amongst participants that the audit tool was not able to measure client engagement, there was discussion about the need to broaden the scope to determine whether unmet standards of care reflected client choice or quality of the service offered, as illustrated:

… so what are you trying to measure, the uptake or also the quality of the care being offered [e.g.: for STI screening], so you’ve got a tick for being offered, but we also know they’re not being screened and if you see, you know 50% of people have… refused it, is it because of the way they’re being offered, so do we need to look at how people are asking/offering that thing, or is it because there is some other thing happening in the community where they don’t want to be part of that?

Participants discussed the scope of the audit eligibility criteria, disagreeing on whether including clients with chronic conditions would bias results. Feedback on the content of the audit tool included comments on the addition of items (main health service attended, chronic conditions care plan), amendments to items (separating diet and physical activity, including internal referrals in referral items) and the deletion of items (medication for eating problems, recording of client information in paper records). At the same time there were some general concerns expressed about the length of the tool.

#### Potential implications for service delivery

The ways in which audit data may inform service delivery was a central aspect of the discussion. Participants discussed how data on clients not attending appointments or declining treatment or referrals may lead to further investigation:

That [clients not attending referral services] might have to be something that as a service we look at…

The potential for data on reports received from referral services to lead to greater communication with external organisations was also discussed:

…there’s very low communication that exists, so that’s what’s designed to help highlight and then you can help prioritise for yourselves if that’s what you want to target, help to increase it…

In the context of a move towards electronic systems and shared electronic health records, this was thought to be particularly pertinent.

Participants discussed how audit data could inform strategies for improved service delivery and outcomes for clients with chronic conditions. The potential for the audit to provide information on their general care including psychosocial screening was highlighted, as illustrated:

…it might tell us whether the care was focussed on that condition rather than being, you know, covering all of the other psychosocial areas, because having done some of the audits that’s what I saw…

#### Quality of documentation in client records

Discussion of the quality of documentation in client records identified some challenging issues. Although it was acknowledged that appropriate documentation was a component of best practice care, there was a concern that some audit items were not well documented and may be difficult to audit. Inconsistencies in the recording of non-clinical items such as referrals, reports received from referral services and mature minor assessments were identified. The potential for the audit to extend to other databases for the purposes of searching for referral information and immunisations was explored.

#### Terminology and definitions

Inconsistent terminology in client records was identified as posing a further potential impediment to the audit process. The challenges of auditing psychosocial assessments were highlighted as participants discussed how to define items in the audit protocol. Greater clarity in definitions of brief interventions, reports received from referral services, long term health conditions, up to date health summaries, clients screened at risk, weight management plans and psychosocial assessments was recommended. Participants generally felt that terminology used within the audit tool should not be overly clinical and be consistent with other One21seventy audit tools for ease of use, particularly by non-clinical staff.

## Discussion

### Tool development and refinement

Consistent with Kötter et al. [21], our process combined extraction of evidence-based guidelines with expert opinion and specific inclusion criteria. An important refinement was our pragmatic approach in seeking advice on the priority audit items and exploring feasibility issues with stakeholders through the ERG and end user focus group processes.

The importance of considering feasibility issues and the need to balance the breadth of items with length were highlighted by both the ERG and user feedback processes. The original One21seventy criteria for developing audit tools (Figure 3) were found to be insufficient for determining items in the youth health audit tool. Further prioritisation of audit items was determined by the ERG according to best practice service delivery for the prevention, screening or treatment for risk factors and conditions identified as priority issues for the health and wellbeing of Indigenous youth and reasonable expectations of health services in the documentation and delivery of care, in addition to the original criteria (Figure 2). ‘Reasonable expectations’ primarily related to the recording of information in client records and the extent of communication and follow up with referral agencies that could reasonably be expected of health services, given resource constraints. It was felt that this approach would enable an adequate assessment of health service performance whilst avoiding setting unattainable standards.

The user feedback process demonstrated the ability of the audit tool to educate health service staff about standards of care and to facilitate reflective practice through both the processes of auditing individual client records and the interpretation of summary results. Some audit items (particularly psychosocial items) were therefore retained in the audit tool despite poor documentation in client records in order to provide opportunities for improvement through staff education and reflection. Appropriate documentation of care is an important component of quality improvement and a legal and ethical issue. Poorly documented items are not graphed in the audit report provided to health services in order to avoid misinterpretation, but presented in tabular format with appropriate commentary on interpretation.

### Limitations

For purposes of brevity and feasibility, the audit tool only assesses guideline-specified prevention, screening and treatment for certain risk factors and conditions prioritised by the ERG. It was not possible to establish criterion validity due to the absence of a ‘gold standard’ comparison to our process [21]. User feedback was only sought through a focus group discussion at one health service, but was combined with auditor notes from pilot testing at four sites and interpreted by the ERG which represented a range of health services. Audit results and inter-rater reliability from pilot testing are not reported in this manuscript as they do not directly relate to its scope but may be reported elsewhere. Further testing of the performance and reliability of the tool is needed.

### Implications

In the context of no articulated standards of care, it is possible to develop an audit process premised on evidence-based guidelines but the justification for each audit item must be explicit and realistic. Our experience was that engagement with stakeholders and experts and the use of selection criteria for audit items were pivotal in determining key measures of quality; and that a pragmatic approach that considered feasibility and reasonable expectations of health services, given constrained resources and the fragmented nature of referral services, was needed. We would recommend that the future development of such tools incorporate these processes and context-specific considerations while allowing further refinement post-implementation.

We expect that audits of youth health service delivery will encourage a greater focus on youth health amongst primary healthcare services, an area often overlooked, and in particular greater attention to screening, assessment and treatment of psychosocial issues. Results from the youth health audit may be of interest to policymakers in designing and supporting primary healthcare delivery models appropriate for Indigenous youth. As there are currently no clearly articulated standards of care specific to Indigenous youth health, policymakers may be interested in the final set of measures included in the audit tool if sector-wide reporting metrics and performance standards are developed for Indigenous youth health in future. Further research is needed to investigate the implementation of the audit tool and the nature and extent of any improvements to the quality of care for youth following successive cycles of use.

## Conclusions

We found that developing robust measures of quality for primary healthcare for Indigenous youth required a pragmatic methodology which was specific, yet flexible. A lack of clearly articulated standards of care, along with gaps and conflicts in guidelines for evidence-based practice, ensured that expert consensus played a fundamental role in determining audit items. Clear criteria were needed to guide expert consensus, but the ability to further refine criteria enabled the Expert Reference Group to respond to issues raised in pilot testing and user feedback processes.

Findings from pilot testing and user feedback processes, that the tool was generally user friendly but impractical in length, facilitated additional prioritisation of audit items by the Expert Reference Group and hence enabled the tool to be further refined. Other feasibility issues raised in these processes, including variable documentation of some services in client records and limits to the ability of practitioners to follow up referral services, highlighted the need for the Expert Reference Group to consider reasonable expectations for health services in documenting and delivering care and avoiding setting unattainable standards, given resource constraints. However, the user testing process also demonstrated how the audit could help educate staff about best practice and provide an opportunity for reflective practice, suggesting a need to balance these functions with the feasibility issues described when establishing performance measures.

To our knowledge, the youth health audit tool represents the first attempt at developing such a resource. Further testing of the performance and reliability of the tool is needed. However, we hope that through incorporation in quality improvement processes such as the One21seventy program, the youth health audit tool will provide a first step in monitoring, assessment and improvement in the way Indigenous primary healthcare services engage with and respond to the needs of youth.
